# The Development of Controlled Orientation of Fibres in SFRC

**DOI:** 10.3390/ma14164432

**Published:** 2021-08-07

**Authors:** Marek Ďubek, Peter Makýš, Marek Petro, Helena Ellingerová, Naďa Antošová

**Affiliations:** Department of Building Technology, Faculty of Civil Engineering, Slovak University of Technology in Bratislava, 810 05 Bratislava, Slovakia; peter.makys@stuba.sk (P.M.); marek.petro@stuba.sk (M.P.); helena.ellingerova@stuba.sk (H.E.); nada.antosova@stuba.sk (N.A.)

**Keywords:** steel fibre reinforced concrete (SFRC), orientation, technical glass beads (TGB)

## Abstract

The article is focused on finding the possibility of the controlled orientation of fibres in fibre reinforced concrete constructions. This is because the controlled orientation of the fibres can contribute to the improvement of some properties of fibre reinforced concrete. The research is based on the experimental investigation of orientation control–rotation of fibres in a transparent matrix representing concrete replacement. From the conceptual model, the article continues with experimentation, data analysis and comparison of conclusions. During the experiment, a mechanical tool was developed and monitored to guide the fibres. The main monitored parameters of the levelling tool were the tips dimensions and the distance between them. The experiment results show the possibility of achieving a higher orientation of the fibres around one axis and suitable parameters of a mechanical tool.

## 1. Introduction

Fibre-reinforced concrete is currently the material used on construction sites. Its advantages lie primarily in blended fibres. The fibres should be homogeneously dispersed. However, their dispersion has a certain shortage. For example, the fibres are in different concentrations at different locations. Additionally, they could be oriented in one place to a greater extent around one of the three axes in 3D space. Fibre orientation in one direction is advantageous for some types of construction. However, orienting the fibres is a difficult process. Therefore, the authors in this article deal with the experimental determination of the controlled orientation of fibres. The controlled orientation of the fibres results in an increase in the initial tensile strength of the fibre reinforced concrete structures. Analysis and calculation methods in the history of this research proved the importance of fibre orientation on the tensile properties of fibre reinforced concrete. The article deals with the results of an experimental investigation of the controlled orientation of fibres. This is based on the optical evaluation of the rotation of the fibre in the transparent matrix. It offers a sufficient basis for continuing research on fibre orientation management. Research methods, as well as results in the field of controlled fibre orientation, are sufficiently presented here.

### 1.1. Steel Fibre of Reinforcement Concrete

The low tensile strength and brittle character of concrete were bypassed by the use of reinforcing rods in the tensile zone of the concrete since the middle of the nineteenth century. The United States was the first to investigate the use of fibres in concrete. At first, they focused only on experiments and later on detailed research. Already at that time, their research clearly confirmed the importance of fibres in concrete. In the beginning, it was the various steel-based segments that were studied [1]. Since then, a substantial amount of research development, experimentation, and industrial application of SFRC-steel fibre reinforced concrete have occurred. In the early 1960s, experiments were carried out using plastic fibres in concrete [2]. The first research was focused on the use of asbestos fibres and later also conventional fibres such as steel and glass. Gradually, research and materials were modernized. Conventional fibres such as steel and glass, as well as new fibres such as Carbon or Kevlar, also proved to be promising. Low modulus fibres, either synthetic (polypropylene, nylon) or natural (cellulose, sisal, jute), have also proven their properties. The fibres were also used in other materials such as plastics, epoxies, and ceramics. In addition to materials, the shape of the fibres gradually developed. Straight ends become curved ends or a wavy shape. The geometry was changed mainly to modify their mechanical connection with the cement matrix.

Currently, there is a recommendation to produce fibre concrete where the recommended dose of steel fibres is between 20 and 40 kg/m^3^. The higher the dose, the greater the flexural strength of the concrete [3]. Fibre dosing is at the end or simultaneously with the aggregate on the conveyor belt [4]. Steel fibres improve the properties of concrete, especially ductility. Compressive strength is slightly affected by the added fibres. Fibres have a greater effect on flexural strength than compressive or tensile strength with an increase of more than 100% [5,6,7] (pp. 471–473).

Fibre-reinforced concrete “SFRC” is a composite material with better qualitative strength characteristics than plain concrete. Thus, the distribution of fibres significantly affects the resulting mechanical properties and performance of the composite [8,9,10,11,12]. All the properties of fibre reinforced concrete are, in real use, able to meet the computing and model requirements, especially when meeting the condition of uniform fibre dispersion. The basic requirement for the full effect of the fibres in the SFRC is uniform fibre dispersion, called homogeneity. The efficiency of the fibres depends very much on their orientation with respect to the geometry and direction of the load and they tend to orient during casting [13] and this tendency has been investigated by many authors, e.g., [14,15,16]. In 1972, a scientist [17] determined the number of fibres intersecting a cross-section by assuming a uniform distribution and applying probability analysis in his paperwork, thus continuing research where experiments analyse the behaviour of fibre reinforced concrete and the number of fibres intersecting a broken cross-section [18,19]. The “orientation factor” generated was derived from the volume of the fibres, the cutting area of the sample and the surface of the cut fibres. It was based on the author of “Fibre Spacing and Specific Fibre Surface” [20].

The author of the research [20] provided a computing method based on empirical results, presented a guiding factor for describing the orientation of the fibres, which became the basis for models of fibre reinforced materials. The orientation factor and its modification was the subject of research by various authors [21,22], who have developed or modified it with regard to the number of fibres, the size of the fibres and their theoretical and actual orientation.

Among other things, the authors later contributed most significantly to the determination of this reference coefficient [9,23,24]. The orientation of the fibres changes in the vicinity of the formwork, and thus the orientation factor also changes, which affects the number of fibres intersecting the cross-section of the fibre reinforced concrete sample. Literature calls this phenomenon wall-effect. Research in the literature was usually focused on beams with a square cross-section. In this case, the wall effect is present on the sides (one side of the form) and in the corners (two sides of the form). The authors [9,25] examined how the form walls affect the orientation factor in the case of steel fibres. In the case of rigid (steel) and flexible (synthetic) fibres, the effect of the formwork on the orientation of the fibres is different, because fibres of different materials [26,27] behave differently with respect to the formwork when in contact with the substrate. This different behaviour will be influenced by its effects on the number of fibres intersecting the cross-section.

### 1.2. The Fibre Orientation

The aim of the research in 2013 [28] was to create a functional model for evaluating and verifying the uniformity of the dispersion of reinforcement in the form of steel fibres. The number of randomly cut fibres incorporated into the work [28] results from the probability of fibres found in cross-section by volume. It is based on the dimensions of the fibres and the recalculation with respect to 1 m^3^ of concrete. Displaying space issue (3D) by means of 2D (surface) image projection is solved by stereology and morphometry, which mean a spatial interpretation of two-dimensional images based on probability and statistics. The created software is able to determine the orientation of the fibres to the x, y and z axes.

In addition to detecting fibre dispersion, it was possible to control their orientation to verify the accuracy of determining fibre orientation. The advantages of controlled fibre direction in the fibre reinforced concrete were also addressed in the literature [21] where the beneficial effects of fibres on tensile properties were found, especially after the cracking. The results were confronted with the number of identified fibres at the crack site [17]. We focused on the properties of fibres anchored to the matrix vertically and at a 30-degree angle. The controlled orientation of the fibres results in an increase in the initial tensile strength of the fibre reinforced concrete structures, as the authors’ simulation calculations had also proved [29]. Similar research of controlled orientation of the fibre impact assessments was already carried out [30]. Analysis and calculation methods proved the importance of fibre orientation on the tensile properties of fibre reinforced concrete.

The behaviour of fibres in fibre reinforced concrete creep was studied by the authors [31,32]. Different fibre reinforced concrete creep rates affect the fibres (final position and rotation) and can cause the fibres to rotate in the same direction as the fibre reinforced concrete material flow. The effect is stronger at a higher flow rate or when the rate may act on the fibre for a longer period of time (i.e., lower viscosity or longer flow time). Thanks to this model, the shape and geometry of the form, the method of filling and the viscosity significantly affect the alignment of the fibres [32,33].

The orientation of the fibres by pouring was also the subject of research. According to this research, fibres that have no obstruction in the creep direction during flowing tend to orient vertically to the creep direction. In addition, besides creeping there is an expansion of fresh fibre reinforced concrete [34,35]. Of course, the orientation is different at the lower edge in the first third of the thickness, in the middle and at the upper edge of the sample. Obviously, the final orientation is also influenced by components such as aggregates. The authors [36] also showed the orientation of the fibres when they were flowing fibre reinforced concrete vertically to the direction, but with a difference at the edges (formwork), which, if slippery, orient the fibres in the direction of creep at the lower edge.

## 2. Materials and Methods

It was necessary to obtain a transparent dense material to simulate fibre reinforced concrete, for which we can change the rheology (mix it). However, after the rheology adjustments do not change the state and the fibres in it will not arbitrarily change positions or rotation. First of all, it is necessary to analyse the composition and structure of concrete. The composition of fibre reinforced concrete can be determined by similar procedures as the design of the composition of plain concrete, including additives and admixtures. The characteristic properties of fibre reinforced concrete, in particular the tensile strength, can be influenced by the type of material used or by the concentration of the dispersed reinforcement. The design must take into account the fact that the composition of fibre reinforced concrete differs from plain concrete. They vary with the fibres added and volume concentrations can in some cases affect the dosages of the components per unit volume of concrete. Then, when designing the fibre reinforced concrete composition, it is necessary to adjust the volumes of the components (aggregate, cement, admixtures) in order to exclude the effect of bulking the aggregate mixture with randomly dispersed fibres.

### 2.1. Aggregates

Aggregate is used as the main strength carrier of concrete. It must have a minimum compressive strength many times higher than the proposed compressive strength of the concrete. Aggregate is a concrete filler made of inorganic substances with grains up to 63 mm. It forms the load-bearing frame of concrete (volume is about 60–80%). According to the design of the type and composition of the concrete, the aggregate gradation curve is also defined. The gradation curve expresses the weight ratio of aggregate under the sieves. Standard sieves have a size in mm: 0.125-0.25-0.5-1-2-4-8-16-32-63. Glass in the form of glass beads, therefore, appears to be a transparent substitute for aggregates.

Glass is an integral part of our environment. It is used in the fields of research, communication technologies, architecture and solar equipment. Glass is an ideal packaging material for beverages, food and cosmetics. Glass is a homogeneous amorphous, isotropic, transparent, solid and fragile substance in a metastable state, formed by cooling the melt. It most often contains silica sand, soda, alkali metal oxides, and limestone. It is a biologically inactive material. The optical properties of glass include refraction of light, dispersion, reflex, transmission, absorption, and colour effect. The mechanical properties of glass include weight, density, elasticity, tensile and compressive strength, brittleness and hardness. The density of the glass beads varies from 2.5 to 4 g/cm^3^.

Glass is a solid amorphous homogeneous, usually transparent substance. It has low thermal conductivity, is relatively resistant to water, gases and other substances. The physical and chemical properties of glass depend on its chemical composition. It is resistant to acids. Use of glass in construction: plate glass (windows), pressed tiles, wall elements, glass fibres (thermal insulation) and many more. Technical glasses are produced by forming (casting, pressing) melting the glass into a product, which is allowed to gradually cool down so that the stress is removed.

**Figure 1:**
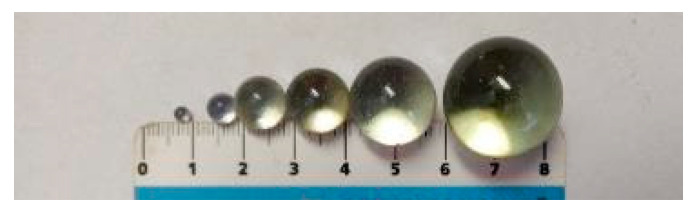
Used technical glass beads.

Glass was used in the research in terms of the issue of obtaining a transparent matrix of concrete, due to its properties described above. The glass thus took over the volume function of the aggregate and formed a matrix. The glass in the experiment was shaped like glass beads. Clear technical glass beads partially met the substitute for aggregates in all fractions, but also clear glass beads refract light and can cause opacity depending on their size. Glass technical “hereinafter referred to as TSG” glass beads with diameters: 0.1–1.3 were selected for the experiment; 3; 6; 10; 12; 16; 22 mm. See Figure 1.

The results of measurements-evaluation of transparency (evaluation was optical based on the number of fibres viewed) for the purpose of verifying the controlled orientation of the fibres are shown in Table 1 and Figure 1. In the case of glass beads with a larger diameter, 80% fibres were identified. With decreasing diameters, the transparency and identification of the fibres reached 0% for dimensions as low as 3 mm and less. Combinations of diameters from 8–16 achieved relatively good transparency and were therefore suitable for monitoring the behaviour of fibres in a controlled orientation simulation. A glass container was used for the simulation, under which a lamp was placed and a camera for shooting was situated on a tripod above the form. It was set to the same distance to ensure identical imaging and evaluation conditions. Only transparencies were observed in the research, as its aim was to retest the controlled orientation of the fibres. In order for the results to be presented immediately after the test, an immediate assessment of the result was required. The glass beads always form a transformable mass, which does not solidify. It is possible to evaluate the rotation of the fibres in it because of the transparency, and it sufficiently represents the concrete matrix.

**Figure 2:**
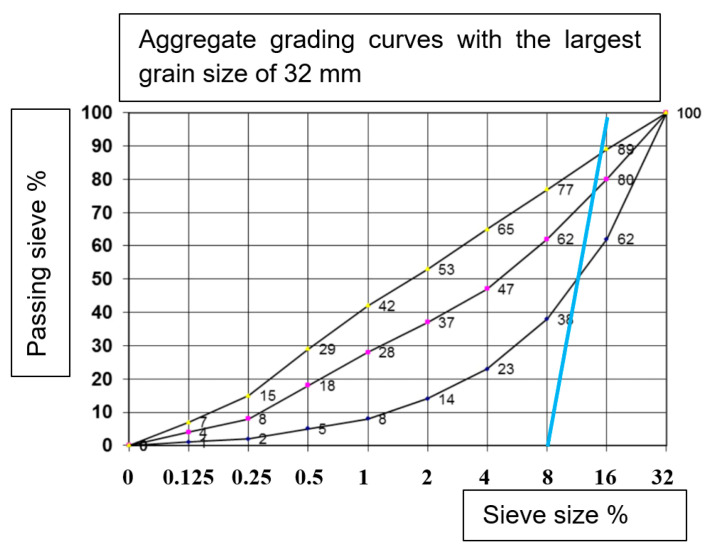
Plotted final gradation curve of technical glass beads—blue line.

The most representative material for forming a transparent matrix is clear glass in the form of glass beads with certain diameters. The amounts of the individual dimensions of the glass beads were designed with respect to the transparency of the test sample mentioned above. The evaluation was performed optically. Based on this investigation, a gradation curve was constructed for the simulation matrix of transparent “concrete”—see Figure 2 below. Transparency was ensured by suitable light and photographic settings. 

The composition of the representative aggregate was based on a visual evaluation of the transparency of the glass beads. The glass beads represent aggregate. Their transparency made it possible to monitor the controlled orientation of the fibres. The aggregate actually used in the concrete could not be replicated to the same dimensions of glass beads. The small beads smaller than 6 mm, did not achieve good transparency. The resulting selected dimensions are selected in the size range 8–16 mm according to Table 1. They showed a transparency between 30–70%.

### 2.2. Fibres

**Figure 3:**
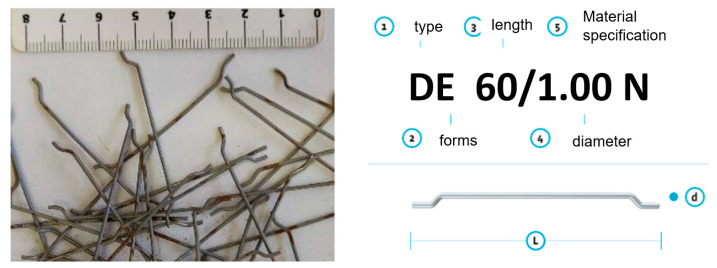
Detail of fibres used in the samples (KrampeHarex, 2020); 1—type; 2—forms; 3—length; 4—diameter of fibre; 5—specify of materials; L—length; d—diameter of fibre.

Steel fibres were used for the research. The type of fibres used was DE 60/1.00 N (KrampeHarex, 2020)—see Figure 3. The fibre manufacturer prescribes in the technical data sheets a minimum dosage of 20 kg/m^3^, a tensile strength of 1100 N/mm^2^ and a modulus of elasticity of 210,000 N/mm^2^. The modulus of elasticity of steel fibres is in the range of 200–210 Gpa. The number (amount) of fibres per j kg is 2700 pcs. The thinness of the fibres (L/d) and the amount of dosage have an effect on the change in the consistency of the fresh concrete after their addition. With the increasing thinness of the fibres at their constant dose, the processing of fresh concrete decreases (fibre reinforced concrete time increase) (Briatka and Ševčík, 2009). Fibre-reinforced concrete thus becomes stiffer, which has an impact on the segregation of aggregate grains and the “sweating of concrete” during compaction. The dose of fibres has a non-linear effect on the change in consistency. 

### 2.3. Transparent Matrix

Observations of the dispersed reinforcement were performed under laboratory conditions. Before the measurement itself, it was necessary to make a form (in the form of an aquarium) for tests with dispersed reinforcement. The basic dimensions of the clear glass form were 600 × 600 × 120 mm. The so-called “aquarium” was mounted on laminated glass, which was supported on the sides so that a light source could be placed under the form. The base laminated glass also fulfilled its load-bearing function, as the filled form weighed 60 kg. An LED light was placed under the form, which made it possible to optically control the dispersing of the fibres. A transparent PVC foil was placed on the bottom wall of the form to protect the glass from the possible impacts of the spikes.

The amount of steel fibres was chosen to be 30 kg/m^3^, taking into account the parameters and recommendations given by the manufacturer and taking into account suitable conditions during testing. The contents of the technical glass beads “Hereinafter also as TSG” were used in the weights given in Table 2.

**Figure 4:**
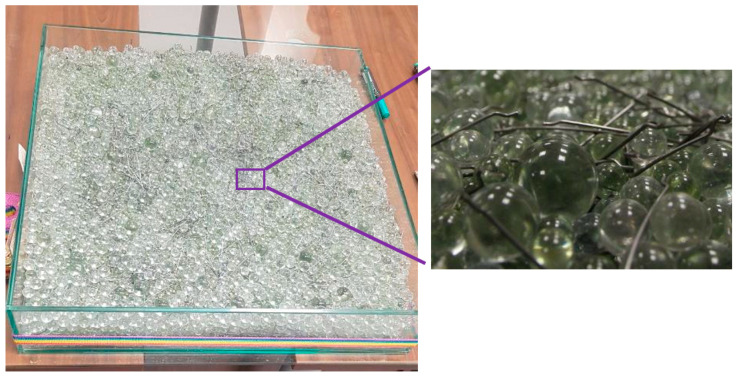
Simulated matrix and detail of fibre reinforced concrete.

The main parameters of the glass form are 600 × 600 × 120 mm completely made of glass with a thickness of 8 mm. The formed volume was 0.036 m^3^. The content of admixed fibres from the basic dosage amount of 30 kg/m^3^ was 1.08 kg (with respect to the volume of the form)—Figure 4. The fibres were weighed and then sufficiently mixed with TSG in a transparent form.

### 2.4. Test—Monitoring the Orientation of Fibres by Creep

**Figure 5:**
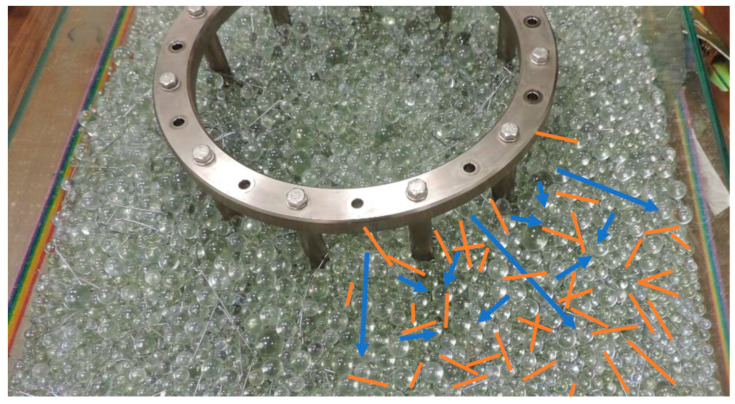
J-ring test monitoring the behaviour of the fibres during creep.

As mentioned above, the fibres tend to rotate according to the flow direction of fibre reinforced concrete, with respect to obstacles and elements, the consistency of the concrete, the roughness of the formed surface, the form walls and of course the vibration. To verify the reliability of the measurement results from the transparent matrix, a test was performed using the J-RING test. The test was to demonstrate the behaviour of the test transparent matrix with fibres and to simulate TSG flow and fibre orientation. Blue arrows—main flow directions and orange lines indicate fibre orientations—Figure 5. Testing and monitoring of the formed transparent matrix with fibres showed the behaviour of the fibres during creep. This means that during creep from one place, when expanding the concrete in the middle layers, the fibres rotate vertically to the direction of creep, at the lower edge they tend to rotate in the creep direction according to the surface roughness of the form and flow off the obstruction. They tend to fill the empty space and orient the fibres according to the creep direction. The main “rectifier” is the coarsest aggregate.

### 2.5. Fibre Orientation Tool

The type of fibres affects the distribution and orientation of the fibres. It was proved that the geometry of structural elements can be a major factor to be considered when designing and controlling fibre orientation. Some fibre orientation patterns had already been simulated, and the formwork surface was found to play a significant role (among other factors) in the final orientation of the fibres immersed in the concrete.

**Figure 6:**
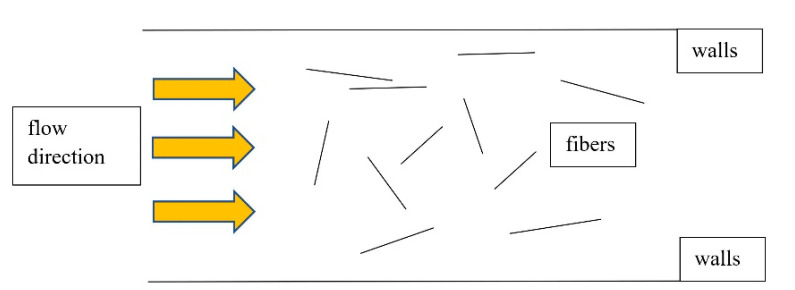
Monitoring of fibre orientation during fibre reinforced concrete creep.

Monitoring the behaviour of the fibres during creep—flow depending on different speeds has already been the research of many authors. Pipe walls were shown to have the most significant effect on fibre orientation. This is followed by the flow rate itself and the structure of the matrix (aggregate). This phenomenon results in a desired or unnecessary orientation. The flow in the formwork itself also causes a controlled orientation, see Figure 6.

The amount of the resistive force is also based on the shape of the flow-preventing element. The most important is how the fluid behind this body manages. The droplet is an example of the lowest resistance [37], which has an ideal aerodynamic shape but is unlikely to tend to rotate the fibres in the direction of its movement.

A levelling tool was made to determine the test-controlled orientation of the fibres. The levelling tool (metal rakes) consists of two 40 × 5 mm strips. The strips are drilled every seven centimetres, with a 7 mm hole for mutual reinforcement with 6 mm screws. Rubber with a thickness of 5 mm is glued to the inner sides of the strips, which prevents the attached tip from moving to the sides. A metal meter is glued to the rubber, which serves for easier orientation during the insertion of the tips. A “t”-shaped handle is welded to one of the strips, which is used to pull the tool. Test tips are placed between the strips and rake out between the glass beads and the dispersed reinforcement.

**Figure 7:**
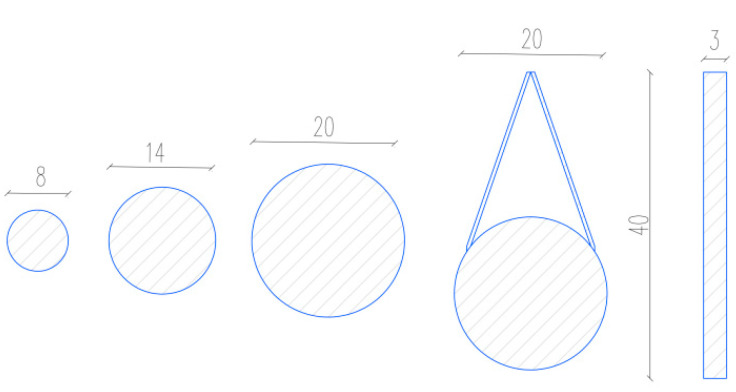
Types of test tips, left: Rod 8; rod 14; rod 20; drop; strips.

A total of 5 types of tips were made for the purposes of the experiment. The three types are logs with diameters of 8; 14 and 20 mm, 200 mm long, at the ends of which the logs are finely ground by means of a lathe. Another type of tip is strips with dimensions of 40 × 170 × 3 mm. The strip is welded at the end to a metal bar so that it can be attached to the tool. As a third type, a representative of the aerodynamic shape and thus the approach to the shape of the droplet was chosen. This was achieved by welding a bar Φ20 mm with two metal plates, in the shape of an arrow. The shapes of the spikes are shown in Figure 7.

**Figure 8:**
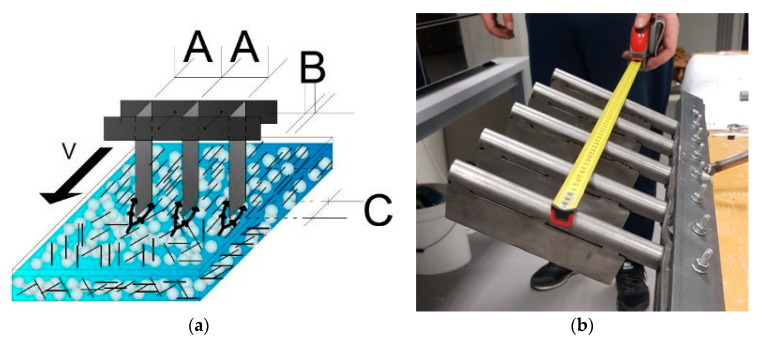
(**a**) Demonstration of controlled fibre orientation research; (**b**) a levelling tool; where: A—Spikes spacing (mm), B,C—Spike type, v—direction of movement.

The purpose of manufacturing this tool in this research was to traverse it between TSGs with steel fibres that simulate fibre reinforced concrete and are transparent Figure 8. The figure shows the device for directing the fibres and its movement direction and the basic monitored parameters: A—spacing of the tips; B—width and shape of the tips; C—length and shape of the tips.

### 2.6. Shooting and Evaluation

**Figure 9:**
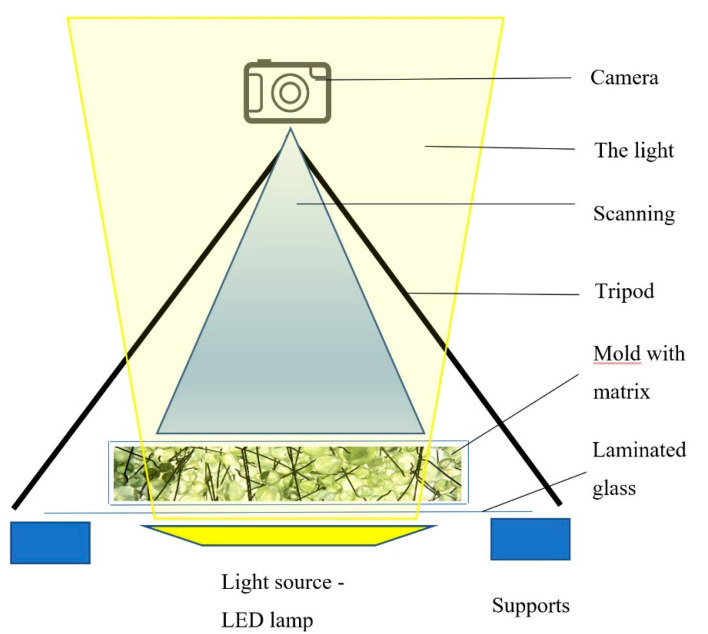
Illustration of the experiment shooting.

For the objectivity of the outcome data, it was necessary to ensure that the photographs were taken under the same conditions and all captured the same part of the form with the simulated material. For this reason, shooting from a tripod placed directly above the form was chosen. The camera was set up to capture primarily the middle part of the form, which was sufficiently backlit by an LED source. Shooting was performed without natural light—only under the light of the source under the form—see Figure 9.

The evaluation was performed optically based on the analysis of photographic images. The aim was to obtain a tool that, with its parameters—dimensions, oriented the most fibres in the direction of its movement. The measurement was evaluated from photos processed after individual measurements, as during the measurements the light illuminated the container and it was impossible for the human eye to recognize the individual wires of the reinforcement, which were deeper. The results are subjective because it is not possible to measure them completely accurately, but it is definitely possible to evaluate them optically and see which results are more favourable. The results evaluated the number of observed fibres and their correct orientation to the ypsilon axis, i.e., to the axis along which the reinforcement was aligned. After each fibre’s orientation, the mixture was mixed and the fibres were homogeneously dispersed.

**Figure 10:**
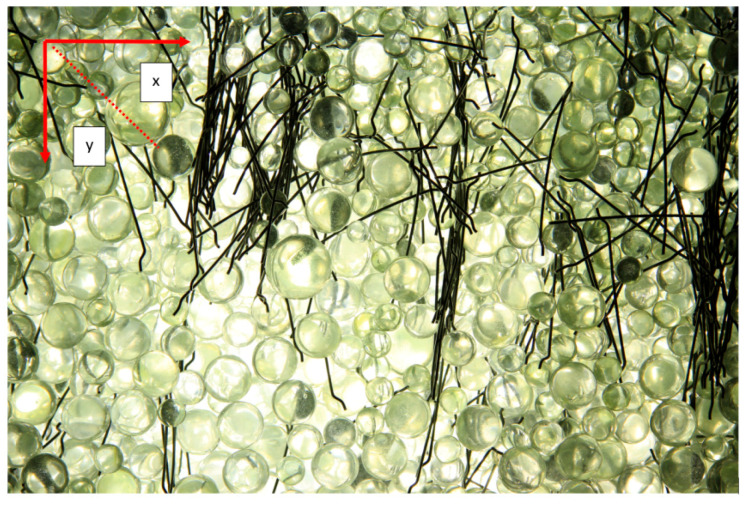
Strips and 2nd move.

The following images show selected images for each tip at the best spacing (90 mm) after the first and second moves. Figure 10 also shows a method of evaluation. This consisted of adding the fibres closer to the “y” axis—in the direction of shift and closer to the “x” axis vertically to the direction of shift. The split angle that determined the assignment to a particular axis was 45°.

## 3. Results

It is clear from the results that if the spacing between the individual tips was 120 mm, then the efficiency of the tool was minimal. The spikes were so far apart that they could not orient the reinforcement correctly. Spacing of fibre width and length (50 to 60 mm) are also completely ineffective. The fibres are unable to pass around the tips and so accumulate in front of them, as there is interaction and agglomeration of the fibres. The fibres thus accumulated also block the path of the TSG, and the tool pushes the entire matrix with the fibres in front of it. Therefore, it is not possible to evaluate the results of such a measurement. At the same time, the amount of pushing matrix multiplies with increasing speed. At low speeds, most of the matrix manages to move behind the tips of the instrument. The most visible results were clearly with spikes spacing of 90 mm (1.5 times the length of the fibres). As expected, the best results in the controlled orientation showed the smallest diameters (thinnest tips) of the tips used. The larger the diameter, the worse the observed results were.

**Figure 11:**
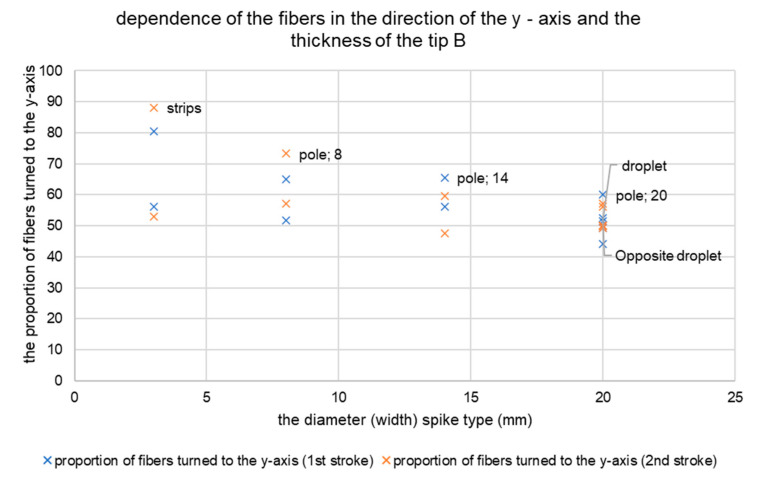

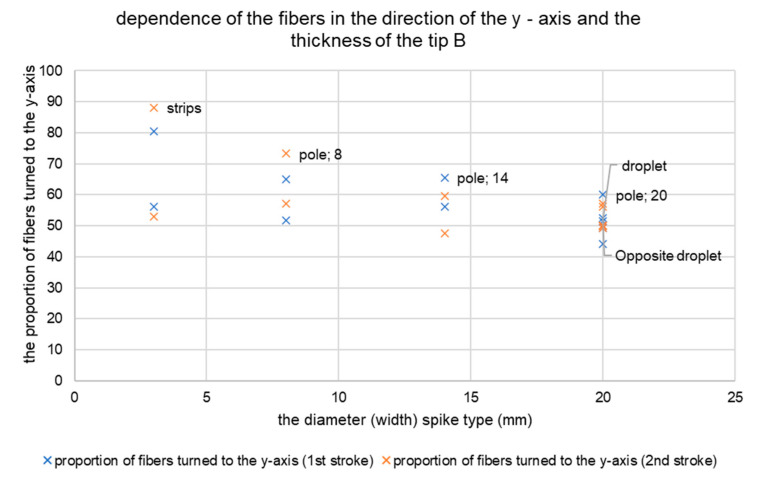
Dependence of fibres in the direction of the *y*-axis and the thickness of the tip B.

The results of the measurements showed a higher concentration of twisted fibres in the direction of the *y*-axis after the second tension with the strip. The strip is the narrowest of the spikes used. After its use, the highest percentage of fibres was rotated in the direction of the *y*-axis, up to 80–90%. The proportion of fibres in the *y*-axis direction was lower in the case of wider tips. The fibres remained relatively homogeneously oriented after the use of wider circular tips. See Figure 11 for the results based on the y-oriented fibres.

**Figure 12:**
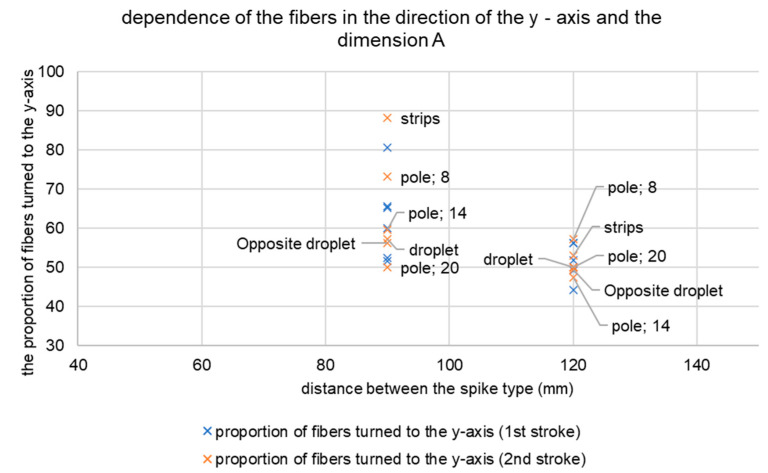
Dependence of the fibres in the direction of the *y*-axis and the dimension A.

In determining the appropriate distance between the tips, 1.5 times the length of the fibre used showed the best results. The distance of 90 mm represents 1.5 times the fibre with a length of 60 mm. They reached a value of 80–90%. Worse results showed spacing of 60 mm. This distance caused the fibres to accumulate and movement to the edge of the form. Therefore, these results are not relevant and are not in the resulting graph. The tip distance of 120 mm did not rotate a sufficient number of fibres in the *y*-axis direction. In the first move and also in the second move it was only 40–60%. The results are shown in Figure 12.

The results showed the need for two moves, with the second being controlled at the location between the tips in the first move. The width of the tip is very important, but it is necessary to minimize its size. It is true that the narrower the tip, the better the rotation of the fibres in the direction of tension. At the same time, the distance between the tips should be 1.5 times the length of the fibre used. The levelling tool assembled in this way achieved the best results and rotated 80–90% of the fibres in the *y*-axis direction.

**Figure 13:**
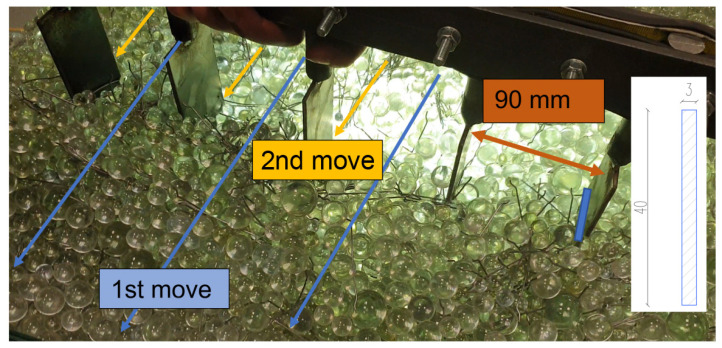
Evaluation of the resulting fibre direction.

The pulling speed of the levelling tool was not monitored, as the rheology of the concrete is different from the test transparent matrix. However, it was possible to observe that the higher the speed, the more fibres were pulled with the levelling tool. Then, it was not possible to evaluate the fibre dispersion. The final design of the levelling tool for the controlled orientation of the fibres is shown in the figure below. The device must be pulled twice—see Figure 13.

## 4. Discussion

Controlled fibre orientation can lead to improved mechanical properties in fibre reinforced concrete structures. Mainly, in the direction of the rotated fibres. This can cause reduced properties in other directions. Fibre-reinforced concrete is currently used in several types of construction. In some of them, controlled fibre orientation is preferred. The filling of the form orients the fibres during filling. All elements placed in the form also affect the orientation of the fibres. Other impacts are the speed of filling the form, the density of the concrete and its consistency. It is also necessary to look for new possibilities of control orientation, which the authors mentioned in the article and which they addressed.

A transparent concrete substitute was used to demonstrate the possibility of controlled fibre orientation. The concrete was replaced by glass technical beads. Steel fibres were used as fibres, which were monitored optically with a light source. The purpose of the optical evaluation was to determine the effect of the tip shape, the spacing between the tips and the speed of movement of the tips for the orientation of the fibres. Testing was based on experimental testing and evaluation of results. The results clearly demonstrate the possibility of controlled fibre direction in fibre reinforced concrete structures. These results will be used in the production of the fibre reinforced concrete sample for further testing.

The use of strips showed the most favourable results. Conversely, the shape of the droplet, which caused the same omnidirectional rotation of the fibres as the circular cross-sections of the tips, was inappropriate. However, the fibres are arranged more slowly according to the arrangement of the glass beads.

To confirm the measurements and control the success of the tip instrument, the measurements were repeated three times. Table 3 presents the results of measurements and optical evaluation of twisted fibres. Fibres around the y- and x-axes were evaluated; fibres to the *z*-axis were not evaluated, as they were not primarily affected by controlled direction, but were affected by rheology during the test secondarily. As the fibres were also relocated in addition to the rotation, the amounts of optically captured and evaluable fibres in the table vary. Only a part of the image area was evaluated, outside the edges of the form, which did not adjust in any way as a result of the test. The width of the test device between the outmost tips was from 350 mm to 360 mm, depending on their amount and the spacing of the tips between them. Therefore, an optical evaluation area of 300 × 300 was obtained.

There were problems with the fibres at the lower edge of the form—about 1/3 of the thickness when it was not possible to clearly identify the fibres. However, it was sufficient to evaluate the orientation of the fibres for these test purposes.

The results showed the need for two moves, with the second being guided between the tips after the first move. The width of the tip is very important, but it is necessary to minimize its size. It is true that the narrower the tip, the better the rotation of the fibres in the direction of tension. At the same time, the distance between the tips should be 1.5 times the length of the fibre used. The levelling tool assembled in this way achieved the best results and rotated 80–90% of the fibres in the *y*-axis direction.

The results are sufficiently representative and it is necessary to produce a fibre reinforced concrete sample with the orientation of the fibres and evaluate their orientation there. That is because the concrete has different properties than transparent glass beads in a matrix. However, the tool is sufficiently capable of orienting the fibres in the desired direction according to the measurements and the results of the experiments.

## 5. Conclusions

This study clearly confirmed the possibility of the controlled orientation of fibres in the concrete. It provides an analysis of the shape of the fibre guide tool. This presented research clearly determined the properties of the device for the controlled orientation of fibres. Its parameters depend on the dimensions of the fibres. This means that the distance between the tips must be 1.5 times the length of the fibres and the dimension of the tip should be in the shape of a rectangle with an aspect ratio of approximately 10:1. The width of the tip is independent, but the use of the device is preferably intended for fibres with a length of 30 to 60 mm. Other fibres have not been tested or researched. The research results provide the first more detailed inputs to the empirical calculations of the static properties of the fibre-reinforced concrete. These input boundary conditions will have the potential in evaluating the properties of fibre concrete. After the production of real fibre concrete with controlled fibre orientation, the results of strength tests will be evaluated. These demonstrate the importance of controlled fibre orientation.

## Figures and Tables

**Table 1:** Test transparency of technical glass beads.

Diameters of Glass Beads-Fraction	Transparency %
22	75
16	70
12	60
10	50
8	30
6	10
3	0
1.0–1.3	0
8–16	65

**Table 2:** Quantities and dimensions of used technical glass beads.

TSG Fraction	Quantity Used in Research (kg)
16	17
12	17
10	17
8	12

**Table 3:** This is a table. Tables should be placed in the main text near to the first time they are cited.

Spikes Spacing (mm)	Diameter (mm)	Spike Type	Axis Rotation y (%) 1. Move	Axis Rotation y (%) 2. Move	1 Move Totally	1 Move to *y*-Axis	1 Move to *x*-Axis	2 Move Totally	2 Move to *y*-Axis	2 Move to *y*-Axis
120			52	57	87	45	42	70	40	30
90	8	pole; 8	65	73	86	56	30	86	63	23
60			x	x	3	1	2	2	1	1
120			56	47	64	36	28	59	28	31
90	14	pole; 14	66	60	58	38	20	42	25	17
60			x	x	6	2	4	4	2	2
120			49	50	63	31	32	60	30	30
90	20	pole; 20	60	50	50	30	20	40	20	20
60			x	x	5	3	2	5	3	2
120			50	50	70	35	35	74	37	37
90	20 × 40	droplet	52	57	62	32	30	70	40	30
60			x	x	5	3	2	4	3	1
120		Opposite droplet	44	49	93	41	52	85	42	43
90	20 × 40		52	56	82	43	39	80	45	35
60			x	x	5	2	3	5	2	3
120			56	53	80	45	35	87	46	41
90	3 × 40	strips	80	88	82	66	16	84	74	10
60			x	x	20	15	5	12	8	4
